# Supplementary material for: Association between oral microbiome and seven types of cancers in East Asian population: a two-sample Mendelian randomization analysis
Source: Front Mol Biosci. 2023 Nov 21;10:1327893. doi: 10.3389/fmolb.2023.1327893 (PMC10702768; doi:10.3389/fmolb.2023.1327893)
Supplement: Supplementary file 1 [file DataSheet1.ZIP › Supplementary Materials/MR plots of saliva/saliva═╝/Prostate cancer/pheno.2608_to_prostate cancer_leave_one_out.pdf]

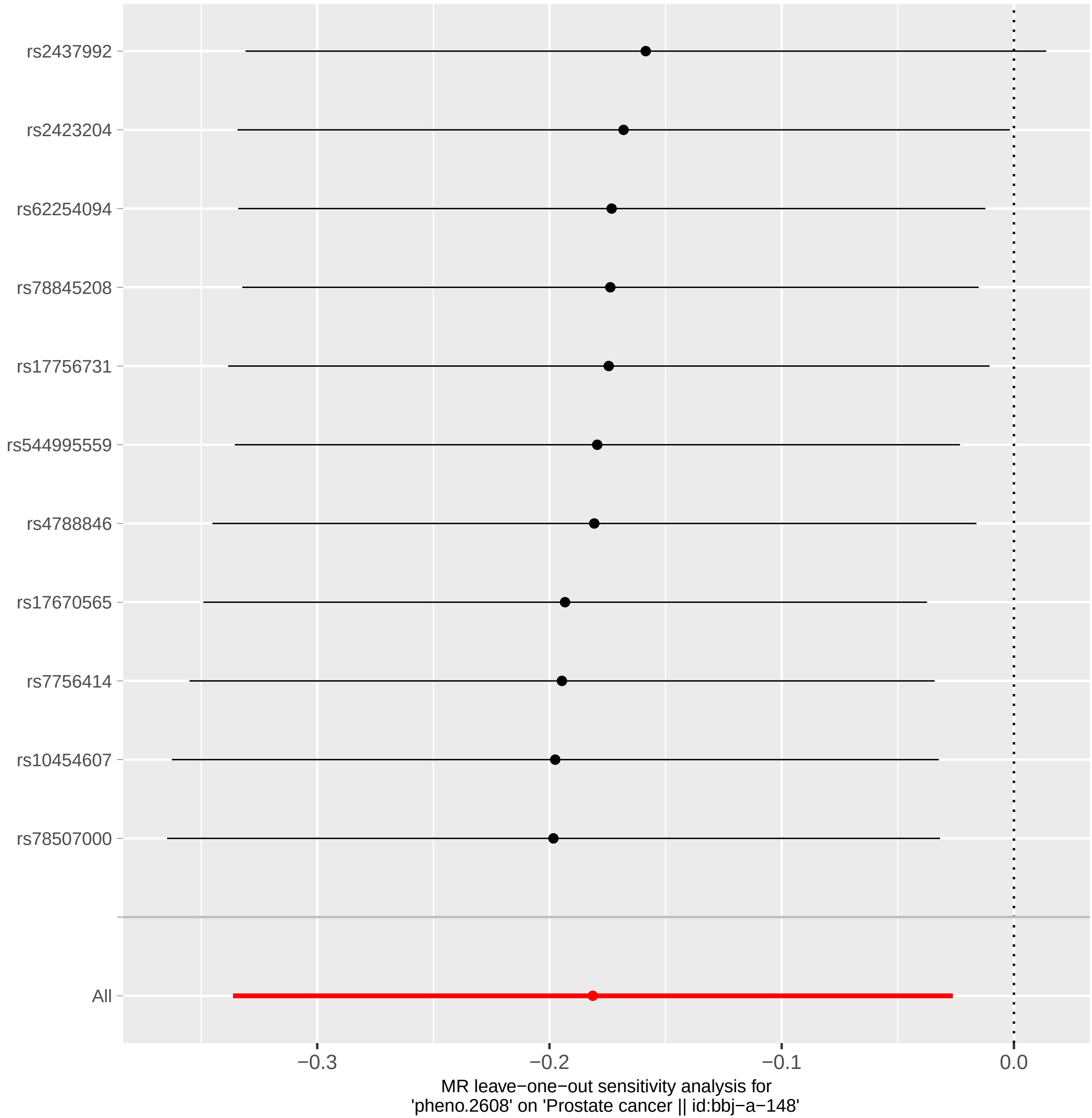

rs2437992
rs2423204
rs62254094
rs78845208
rs17756731
rs544995559
rs4788846
rs17670565
rs7756414
rs10454607
rs78507000
All
−0.3
−0.2
−0.1
0.0
MR leave−one−out sensitivity analysis for
'pheno.2608' on 'Prostate cancer || id:bbj−a−148'
